# Current Protections and Future Threats to Say's Spiketail Habitat in the Southeastern USA

**DOI:** 10.1002/ece3.73656

**Published:** 2026-05-08

**Authors:** J. Matthew Flenniken, Mark A. Barrett, Dirk J. Stevenson

**Affiliations:** ^1^ Fish and Wildlife Research Institute Florida Fish and Wildlife Conservation Commission Gainesville Florida USA; ^2^ Fish and Wildlife Research Institute Florida Fish and Wildlife Conservation Commission Tallahassee Florida USA; ^3^ Altamaha Environmental Consulting Hinesville Georgia USA

**Keywords:** *Cordulegaster sayi*, gap analysis, Maxent, Odonata, species distribution model, *Zoraena sayi*

## Abstract

Dragonflies and damselflies are important indicators of ecological integrity and environmental quality but remain underrepresented in conservation efforts, in part due to a lack of quantitative information regarding their habitat requirements. Species distribution models (SDMs) can facilitate conservation planning by allowing researchers to estimate habitat suitability, evaluate conservation status, and quantify future threats. Using Maxent, we developed a range‐wide SDM for an imperiled North American dragonfly ( *Zoraena sayi* ) to estimate the extent and distribution of potential breeding habitat. To capture the full spectrum of habitats used by *Z. sayi* across their multi‐stage life cycle, we additionally extended the predicted area to include potential terrestrial habitat used by adults within the surrounding landscape, thereby producing a holistic model of breeding (nymph) and terrestrial (adult) habitats. We then used this combined model to quantify coverage by existing protected areas and evaluate projected impacts from urbanization. We found that of the combined model area (2731 km^2^), only 30% is safeguarded by existing protected areas, with 50% of the remaining unprotected habitat projected to experience impacts from urbanization by 2070. Our model represents the first comprehensive assessment of suitable habitat for *Z. sayi* and may be useful in guiding conservation planning aimed at effectively safeguarding suitable habitat and preserving remaining populations.

## Graphical abstract

Dragonflies and damselflies are important indicators of ecological integrity and environmental quality but remain underrepresented in conservation efforts, in part due to a lack of quantitative information regarding their habitat requirements. Species distribution models (SDMs) can facilitate conservation planning by allowing researchers to estimate habitat suitability, evaluate conservation status, and quantify future threats. Using Maxent, we developed a range‐wide SDM for an imperiled North American dragonfly ( *Zoraena sayi* ) to estimate the extent and distribution of potentially suitable habitat and subsequently evaluated current conservation protections and future threats from projected urbanization. We found that a large proportion of potentially suitable habitat is currently unprotected and vulnerable to future urbanization, highlighting opportunities for conservation moving forward.

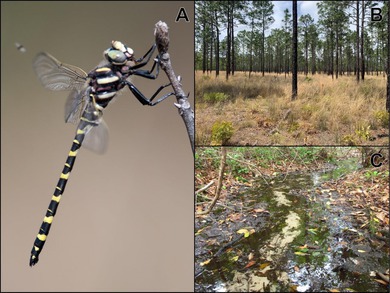

## 1 Introduction

Dragonflies and damselflies (Order: Odonata) link freshwater and terrestrial ecosystems through the completion of their multi‐stage amphibious life cycle and are considered indicators of ecological integrity and environmental quality in both wetland and riparian ecosystems (de paiva Silva et al. 2010; Kutcher and Bried 2014; Miguel et al. 2017; Gómez‐Tolosa et al. 2021). Despite their significant ecological value and wide representation in ecological research, dragonflies and damselflies remain underrepresented in conservation planning and receive limited protection in the United States, Europe, and elsewhere (Bried and Mazzacano 2010; Tang and Visconti 2021; Samways et al. 2025). A global assessment of Odonata by Clausnitzer et al. (2009) concluded that one in 10 species is at risk of extinction.

Species distribution models (SDMs) are valuable tools for conservation planning (Villero et al. 2017; Franklin 2023). SDMs can be used to predict species' distributions (Rosner‐Katz et al. 2020), evaluate conservation status (Bosso et al. 2018), and quantify future threats (Barrows et al. 2010). Selecting appropriate and informative environmental covariates is essential when building SDMs, and the spatial scale at which covariates are incorporated can influence their predictive accuracy, particularly for species with specialized habitat requirements (Matutini et al. 2021). Multi‐scale SDMs, which incorporate covariates at multiple spatial scales, consistently outperform single‐scale models (Hallman and Robinson 2020) and are therefore recommended when modeling the distributions of dragonflies and damselflies (Collins and McIntyre 2015).

Modeling the ecological niche of an organism with a complex life history presents unique challenges. For example, adult dragonflies are capable of long‐distance dispersal, and, as such, their presence is not necessarily indicative of suitable breeding habitat (Raebel et al. 2010; Patten et al. 2015). Presence of nymphs (aquatic larvae) or their exuviae (shed exoskeletons) provide evidence of successful reproduction, but their low detectability can result in an underestimation of occurrence (Bried et al. 2012) and an overall lack of data. Patten et al. (2015) found that SDMs built exclusively with adult occurrence records differed significantly from those built with records indicating breeding (i.e., based on tandem pairs [adults in copula], ovipositing [egg‐laying] females, nymphs, teneral [recently emerged] adults, or exuviae). Specifically, adult‐only models predicted broader geographic distributions and less‐specialized environmental niches. However, for riverine species, such overgeneralization can be mitigated by moving adult occurrences downslope to the nearest stream reach, to thus restrict presence records to potential breeding habitat (Collins and McIntyre 2017). Furthermore, observations submitted by community scientists (which tend to be adult‐biased for Odonata, Collins and McIntyre 2017) can be included to supplement low sample sizes in traditional survey data (Feldman et al. 2021; Matutini et al. 2021). However, perhaps due to the dearth of readily available occurrence data relative to other major insect orders (Feldman et al. 2021), the distributions of many of the world's Odonata remain unexamined (Collins and McIntyre 2015), including for species of conservation concern.

**FIGURE 1:**
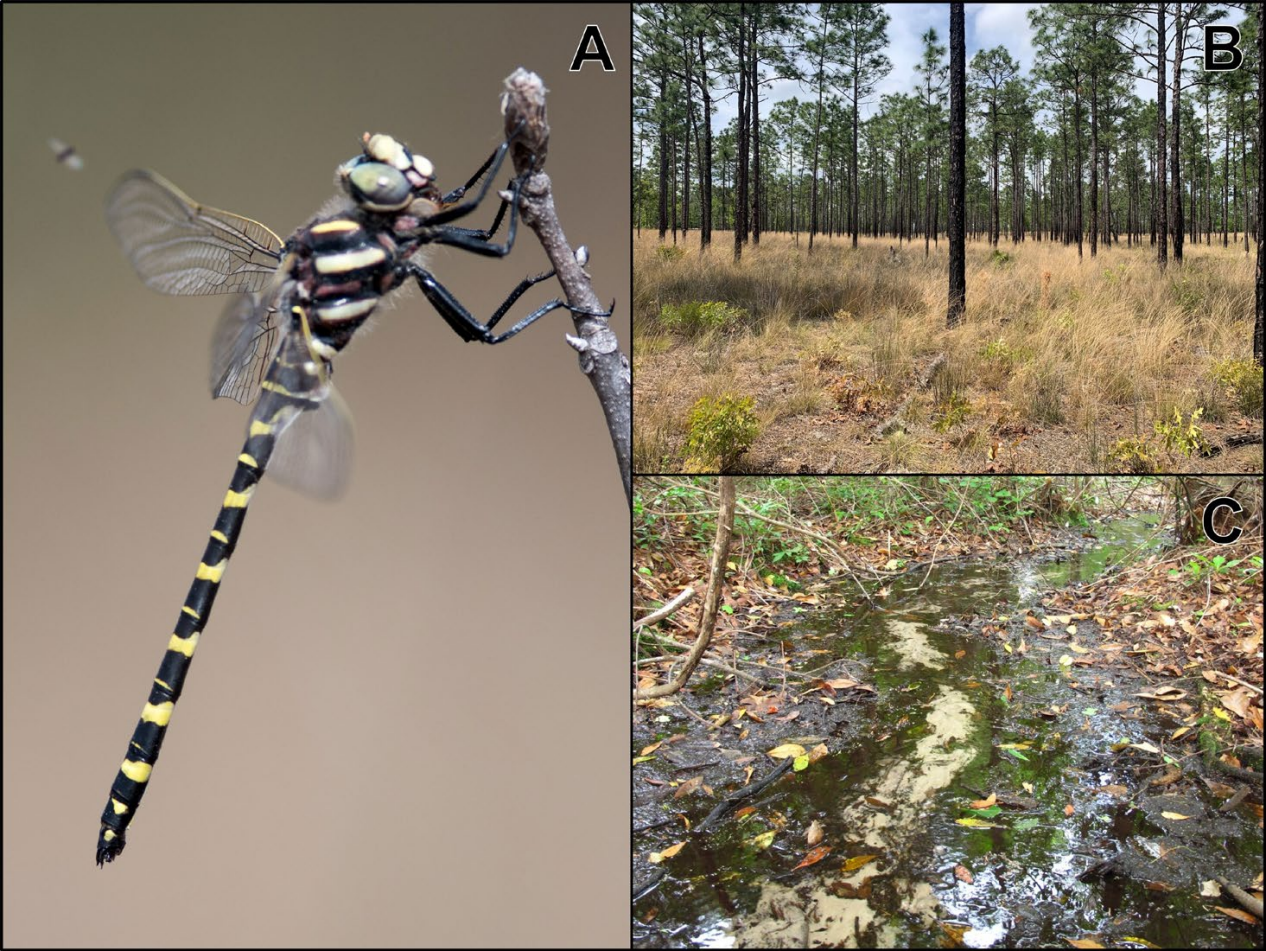
*Zoraena sayi* breeding and terrestrial habitat. (A) An adult male *Z. sayi* . Picture credit: Jonathan D. Mays. (B) Typical habitat used by adult *Z. sayi* —a fire‐adapted, xeric upland community dominated by longleaf pine ( *Pinus palustris* ), turkey oak ( *Quercus laevis* ), and wiregrass ( *Aristida stricta* ). Picture credit: J. Matthew Flenniken. (C) Typical habitat used by nymphs and breeding adults—a perennial forested seepage embedded within/downslope of xeric uplands. Picture credit: Dirk J. Stevenson.

*Zoraena sayi* Selys (Say's Spiketail), formerly *Cordulegaster sayi* , is a species of dragonfly endemic to the coastal plains of the southeastern United States. Nymphs are associated with first‐ to second‐order perennial forested seepages embedded within or downslope of fire‐adapted, xeric upland communities dominated by longleaf pine ( *Pinus palustris* ), turkey oak ( *Quercus laevis* ), and wiregrass ( *Aristida stricta* ), where adults are known to forage (Figure 1; Stevenson et al. 2009; Keppner 2013). Longleaf pine forests, once widespread throughout the region, have declined in acreage by roughly 98% in the last century (Noss and Scott 1995; Frost 2006; Florida Natural Areas Inventory 2010). Owing to the historical loss and continued vulnerability of these habitats to destruction, fragmentation, and degradation, *Z. sayi* is a species of conservation concern in Florida (Florida Fish and Wildlife Conservation Commission 2019) and Georgia (Penland et al. 2025). Although the geographic range of *Z. sayi* has been delineated (Paulson 2011), its habitat distribution has not been estimated, nor have threats to its habitat been quantified on a large scale.

In the southeastern United States, anthropogenic land use change, specifically from urban development and logging, represents a potential threat to *Z. sayi* populations (Paulson 2018). As human populations in the region continue to grow (U.S. Census Bureau 2024), anthropogenic impacts to *Z. sayi* habitat are likely to increase. Human‐induced disturbances that degrade wildlife habitat often result in geographic range contraction and subsequent population declines (Channell and Lomolino 2000). Dragonfly populations can be impacted by a variety of direct and indirect disturbances to their breeding habitats. For example, silviculture operations that harvest timber near breeding habitats are often associated with reduced abundance and diversity of dragonfly assemblages (Calvão et al. 2016; Luke et al. 2017). Furthermore, freshwater habitats within intensive agricultural landscapes are consistently less suitable for breeding dragonflies (rare and common species alike) than those within less‐impacted landscapes (Collins and McIntyre 2017; Baeta et al. 2024). While protected areas can mitigate some direct anthropogenic impacts, habitat degradation may still occur due to human activities (e.g., mining, logging, ranching) within areas designated for multiple uses, or from indirect impacts (e.g., runoff, pollution, contamination) from adjacent land uses (Chape et al. 2005). Nevertheless, protected area networks are essential for the conservation of many species, including *Z. sayi* .

Our objective was to estimate the extent and distribution of potential habitat for *Z. sayi* , identify current conservation gaps (i.e., where potential habitat is not covered by existing protected areas), and gauge the projected future impacts due to land use change. Using Maxent (Phillips et al. 2006), we developed a multi‐scale SDM to predict potential breeding habitat for *Z. sayi* throughout the entirety of their range in the southeastern United States. To capture the full spectrum of aquatic and terrestrial habitats required to complete their life cycle, we additionally extended the predicted area to include potential terrestrial habitat used by adults within the surrounding landscape, thereby producing a holistic model of breeding (nymph) and terrestrial (adult) habitats. Using this model, we quantified contemporary conservation gaps and evaluated the potential impacts of projected urbanization. By evaluating current protections and future threats to *Z. sayi* habitat, we hope to inform targeted conservation actions aimed at safeguarding suitable habitat and preserving existing populations.

## 2 Methods

### 2.1 Study Area

**FIGURE 2:**
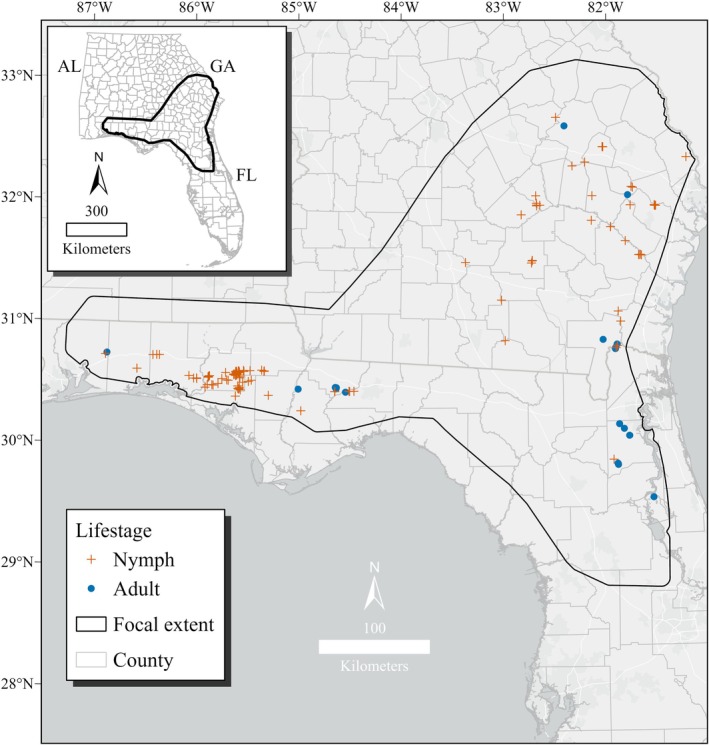
Final set of occurrence records (*n* = 132) used to model breeding habitat for *Zoraena sayi* in the southeastern United States. Data were collected from 2008 to 2024 in Florida and Georgia. Focal extent was based on estimated *Z. sayi* range from Paulson (2011) plus a 10 km buffer.

Our study focused on the entire known range of *Z. sayi* , as described in Paulson (2011). We added a 10‐km buffer to the perimeter of the range to accommodate edge‐of‐range occurrence records; this defined the boundaries of our model and analyses. Accordingly, our study area was 117,423 km^2^ across portions of Florida (42%), Georgia (55%), and Alabama (3%; Figure 2). This area lies within the Southeastern Plains and Southern Coastal Plain ecoregions of North America (Omernik and Griffith 2014)—regions once dominated by longleaf pine forests, but now mostly converted to tree plantations, agricultural lands, and urban areas (Sayler et al. 2016). Upland forests (e.g., remnant longleaf pine forests, sandhills, and savannas), forested wetlands, and freshwater streams comprise the majority of remaining natural communities within the study area (Dewitz 2021).

### 2.2 Occurrence Data

We obtained 211 occurrence records of *Z. sayi* nymphs (*n* = 128) and adults (*n* = 83) collected between 2008 and 2024. Sources included targeted field surveys in Florida (Keppner 2013; Flenniken, J. M. [2025], unpublished data) and Georgia (Stevenson et al. 2009; Stevenson 2014), databases maintained by the Florida Natural Areas Inventory and Georgia Department of Natural Resources, and opportunistic sightings compiled from community science databases (iNaturalist and Odonata Central). We reviewed every occurrence record for accuracy and removed duplicate records and those with obvious spatial errors. For occurrence records from iNaturalist, only Research Grade observations with open geoprivacy settings were retained. Following the methods of Collins and McIntyre (2017), we moved adult occurrences downslope to the nearest stream reach (within 300 m) to mitigate the influence of terrestrial habitat associations and restrict presence records to potential breeding habitat. Then, to avoid pseudoreplication due to sampling bias, we spatially thinned all occurrence data such that only one record was associated with any given stream reach. If both nymph and adult records were associated with a stream reach, we retained the nymph record. If multiple records of the same life stage were present, a single record was chosen randomly.

### 2.3 Environmental Data

Based on prior research (Stevenson et al. 2009; Keppner 2013), professional opinion (J. D. Mays, personal communication), and direct field observation, we compiled 12 potentially important environmental variables that characterize climate, land cover, soil, and topography, at local, catchment, and regional scales (Table 1). To account for differences in spatial resolution between environmental variables and accommodate the spatial uncertainty associated with occurrence records, we converted each raster dataset to 60‐m resolution using bilinear interpolation in ArcGIS Pro 3.3.2 (Esri, Redlands, CA, USA). To build some variables, we applied circular neighborhoods (i.e., focal windows). The optimal size of focal windows used in SDMs can vary based on landscape features (e.g., edaphic vs. topographic) and species size and mobility (Collart et al. 2025). To account for potential scale‐dependent relationships between environmental variables and species presence across multiple life stages, we explored using 60‐m, 120‐m, and 540‐m radii to summarize values for the center pixel in each neighborhood. These radii roughly correspond to the scale of habitat use by nymphs (60 m) and adults (120–540 m; Flenniken, J. M. [2025], unpublished data). We determined the 120‐m radius to be optimal based on weight‐of‐evidence and information values using the Information package (Kim 2025) in R 4.3.2 (R Core Team 2022). We ran pairwise collinearity tests among the 12 variables using the Pearson correlation coefficient (*r*) for continuous variables and the ANOVA correlation ratio (*η*) for categorical variables. We applied a threshold of 0.70 but found no significant relationships (*r* ^2^ or *η* ^2^ ≤ 0.39 for all pairwise tests).

**TABLE 1:** Environmental variables (*n* = 12) used to model breeding habitat for *Zoraena sayi* in the southeastern United States. Variables characterize climate, land cover, soil, and topography at local, catchment, and regional scales.

Variable	Category	Scale	Native resolution	Description
Slope^a^	Topography	Local	30 m	Mean slope within a 120‐m circular neighborhood
TPI^a^	Topography	Local	30 m	Slope position within a 120‐m circular neighborhood
Landform^a^	Topography	Local	30 m	Geomorphon classes (*n* = 10)
Conductivity^b^	Soil	Local	10 m	Mean soil conductivity within a 120‐m circular neighborhood
pH^b^	Soil	Local	10 m	Mean soil pH within a 120‐m circular neighborhood
% Sand^b^	Soil	Local	10 m	Mean percent sand within a 120‐m circular neighborhood
Distance to stream^c^	Land Cover	Local	30 m	Distance to headwater flowlines (Strahler Orders 1 and 2)
Distance to terrestrial habitat^c^	Land Cover	Local	30 m	Distance to Evergreen Forest, Shrub/Scrub, and Grassland/Herbaceous land cover classes with < 50% canopy cover
% Developed^c^	Land Cover	Catchment	30 m	Percent developed within subwatershed (HUC 12)
% Agriculture^c^	Land Cover	Catchment	30 m	Percent agriculture within subwatershed (HUC 12)
Temperature (March)^d^	Climate	Regional	4 km	Mean temperature for March from 2010 to 2024
Precipitation (March)^d^	Climate	Regional	4 km	Mean precipitation for March from 2010 to 2024

^a^ Computed from National Elevation Dataset (USGS 2024a).

^b^ Soil Survey Geographic Database (SSURGO; NRCS 2025).

^c^ Computed from National Land Cover Database 2019 (Dewitz 2021).

^d^ PRISM (Daly et al. 2000).

At large spatial scales (i.e., the scale of a species' entire geographic range), climate conditions strongly influence the geographic distribution of dragonflies (Pearson and Dawson 2003; Collins and McIntyre 2017). As dragonflies are ectotherms, air and water temperature (which are highly correlated; Morrill et al. 2005; Caissie 2006) govern important aspects of physiology, including development (Pritchard and Leggott 1987) and reproduction (Minot et al. 2021). Likewise, precipitation may influence dragonfly distributions through its impact on hydrology (Ball‐Damerow et al. 2014). To produce climate variables, we used PRISM data (Daly et al. 2000) downloaded from ClimateEngine.org. We quantified mean temperature and precipitation for March (when most adult *Z. sayi* emerge) from 2010 to 2024.

Land cover affects habitat suitability for dragonflies both directly, by influencing adult movement and thermoregulation at local scales (Dolný et al. 2014; Minot et al. 2021), and indirectly, through the impacts of hydrological inputs at the catchment scale on breeding and nymphal habitat (Collins and McIntyre 2017; Baeta et al. 2024). To produce land cover variables, we used land cover data and tree canopy cover data from the National Land Cover Database (NLCD) for 2019 (Dewitz 2021). Because our occurrence data spanned 16 years (2008–2024, with 85% of records from 2013 to 2024), we used land cover data from an intermediate year (2019) to mitigate possible effects due to changes in land cover over time (White et al. 2024). Additionally, we evaluated land cover change from 2008 to 2024 across the entire study area and at the location of each occurrence record. To accomplish this, we downloaded NLCD land cover data for 2008, 2019, and 2024. For each land cover class, we computed the change in total area and relative percentage area (1) across the study area.

(1) $$\left[\left(A_{1,\mathrm{ti}+1}/\sum_{i=1}^{n}A_{i,\mathrm{ti}+1}\right)-\left(A_{1,t1}/\sum_{i=1}^{n}A_{i,t1}\right)\right]\times 100$$

If a change in land cover class was detected at the location of an occurrence record, we cross‐referenced satellite imagery (Google Earth Pro 7.3.7) to determine whether the change was genuine or merely resulting from variance in mapping processes (e.g., resolution or classification errors, salt‐and‐pepper noise). To produce catchment‐scale variables, we computed the percent developed area (Developed—Low, Medium, and High Intensity) and percent agricultural area (Pasture/Hay and Cultivated Crops) within subwatersheds (12‐digit hydrologic unit codes [HUC]; U.S. Geological Survey [USGS] 2025b). To represent potential terrestrial habitat, we computed the distance from Evergreen Forest, Shrub/Scrub, and Grassland/Herbaceous land cover classes with < 50% canopy cover based on the NLCD tree canopy cover dataset.

Hydrological conditions, including water chemistry, influence habitat suitability for dragonflies and other freshwater invertebrates (Booker et al. 2015; Perron and Pick 2020). However, national coverage of water quality data is not readily available for many semi‐permanent or low‐flow watercourses, such as the seepage systems used by breeding *Z. sayi* . In the absence of these data, local‐scale soil characteristics and topographical features can be useful predictors of geographic distribution and habitat suitability due to their indirect influence on hydrological variables like water velocity and O_2_ content (Collins and McIntyre 2015). Additionally, as many dragonfly nymphs exhibit strong preferences for specific substrates, soil characteristics may be informative in their own right (Leipelt and Suhling 2001). To produce soil variables, we used data from the Soil Survey Geographic Database (SSURGO; National Resources Conservation Service [NRCS] 2025). We extracted three soil attributes (Electrical Conductivity, pH, and Percent Sand) and used a 120‐m circular neighborhood to compute the mean value of each attribute per pixel. To produce topographic features, we used a digital elevation model (DEM) from the National Elevation Dataset (U.S. Geological Survey [USGS] 2024a). We computed the mean slope of pixels within a 120‐m circular neighborhood and produced a topographical position index (TPI) representing the relative elevation of a location to its surroundings by subtracting the mean elevation within the neighborhood from the elevation of each pixel. Positive TPI values indicate ridges and negative TPI values indicate valleys. To identify terrain features, we used the Geomorphon Landforms tool in ArcGIS Pro to produce a categorical landform variable with 10 classes: 1. Flat, 2. Peak, 3. Ridge, 4. Shoulder, 5. Spur, 6. Slope, 7. Hollow, 8. Footslope, 9. Valley, and 10. Pit. We used a search distance of 10 pixels and a skip distance of 3 pixels (to smooth the landform classification).

The seepage systems used by breeding *Z. sayi* were not adequately represented by the flowlines in the National Hydrography Dataset (NHDPlus HR; USGS 2025a) based on data exploration of occurrences. To produce more detailed stream reaches and identify headwater flowlines nearer to occurrence records, we used the Hydrology toolset in ArcGIS Pro. We classified flow accumulation from the DEM using a relatively low value of 300. We determined the stream order of the modeled flowlines and retained only headwater segments with Strahler Orders 1 and 2. As producing these high‐resolution flowlines tended to create many artifacts (i.e., lines not related to streamflow networks), we cleaned the data using a rules‐based technique to reduce noise. First, we omitted flowlines that occurred within waterbody interiors and removed long straight‐line segments (e.g., sunburst/fan artifacts) with sinuosity values ≤ 1.01. We retained modeled flowlines that occurred within 3 km of NHDPlus HR flowlines based on the mean + 2 SD (i.e., removed outliers) distance to our modeled flowlines. From these reduced flowlines, we retained those that occurred in landform classes 6–10 (potential stream valleys) and were connected to Woody Wetlands. Finally, we removed isolated short segments that were < 100 m long and > 1.5 km from other flowlines. We used the remaining high‐resolution flowlines to produce a distance‐to‐stream variable and define the environmental space during model development.

### 2.4 Model Development

**FIGURE 3:**
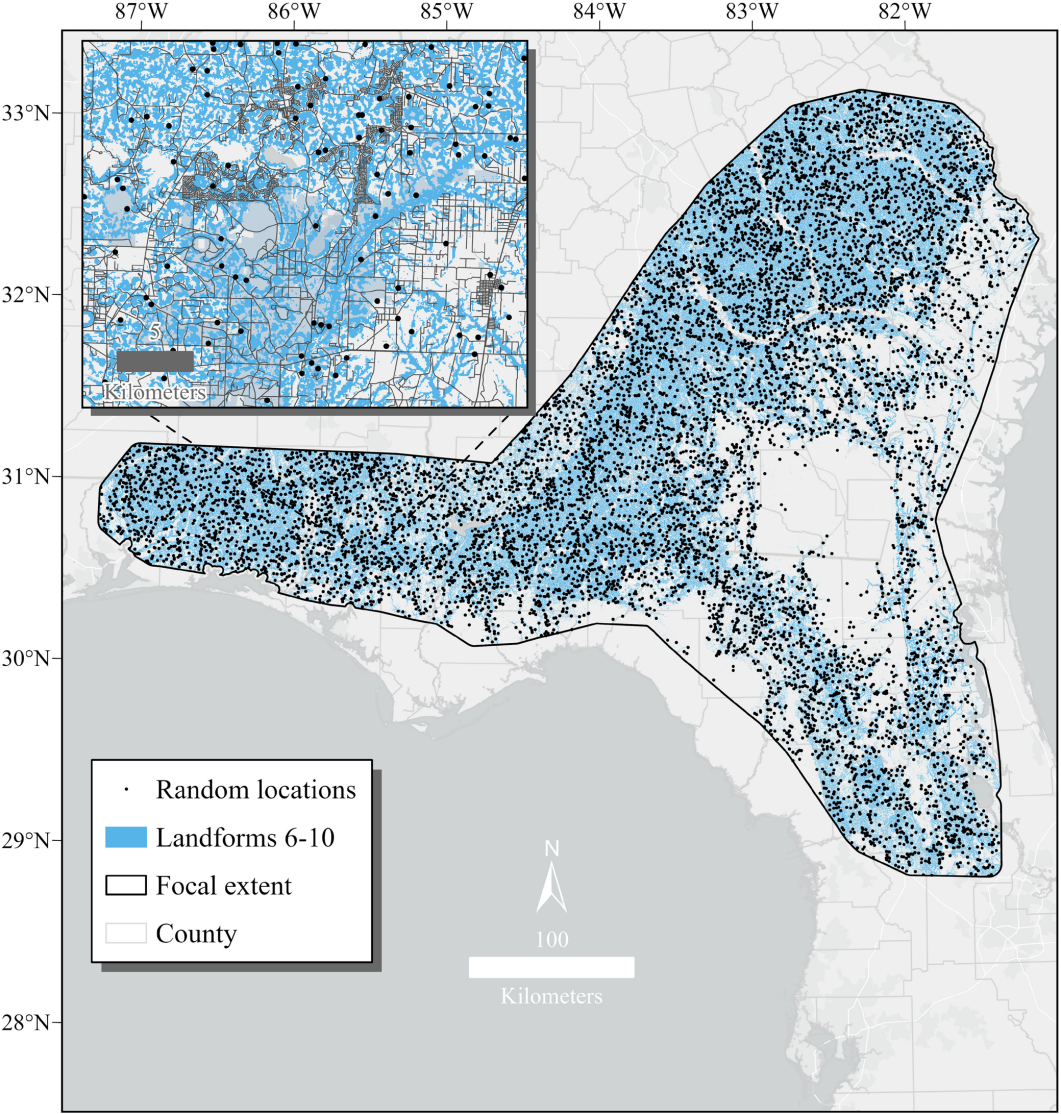
Random background locations (*n* = 10,000) used to model breeding habitat for *Zoraena sayi* in the southeastern United States. To account for sampling bias associated with occurrence records, background points were distributed near landform classes 6–10 (used to generate high resolution flowlines) and roads (inset map), matching the biased distribution of occurrence records. Focal extent was based on estimated *Z. sayi* range from Paulson (2011) plus a 10 km buffer.

We used Maxent 3.4.1 (Phillips et al. 2006) to predict potential breeding habitat for *Z. sayi* . We first conducted hierarchical model selection using the ENMeval package (Kass et al. 2021) in R to determine optimal model parameters based on the lowest Corrected Akaike Information Criterion (AICc) score of the candidate models. Model parameters assessed included the regularization multiplier and feature classes (linear, quadratic, hinge, and product [LQHP]); we excluded the threshold (T) feature because it was replaced by the hinge feature and tends to overfit to training data (Phillips et al. 2017). We defined landform class as a categorical feature. We used the default of 10,000 random background points to capture the environmental space. To evaluate the model, we split the presence and background data into training (90%) and test (10%) bins using 10‐fold cross‐validation. We averaged the 10 model runs to produce likelihood scale (0–1) habitat maps using complementary log–log (cloglog) output (Phillips et al. 2017). As skewed sampling can bias model outputs and commonly reported evaluation metrics (Lobo et al. 2008), we generated random background points from landform classes 6–10 (potential stream valleys) to match the distribution of presence locations (Phillips et al. 2009; Figure 3). Additionally, because a significant portion of occurrences were near roads (approximately 30% ≤ 100 m), likely due to observer bias, we used a distance function to proportionally distribute the background points nearer to roads (Figure 3).

### 2.5 Model Evaluation

Careful evaluation of model performance is essential to the utility and reliability of SDMs (Vaughan and Ormerod 2005). A variety of evaluation metrics are commonly implemented, each with their respective limitations (Allouche et al. 2006; Lobo et al. 2008; Wunderlich et al. 2019). To avoid reliance on any single metric, we evaluated our model using a combination of quantitative and qualitative approaches.

We first evaluated the model using receiver‐operating characteristic curves (ROCs) and their associated area under the curve (AUC) scores. We evaluated model performance with the training AUC and model predictability with the test AUC. We computed the mean and standard deviation of AUC scores across the 10‐fold model runs and classified scores as outstanding (> 0.90), excellent (0.80–0.90), acceptable (0.70–0.79), poor (0.50–0.69), or invalid (< 0.50) based on Hosmer et al. (2013). We determined whether the model was overfitted to the data by qualitatively comparing the training AUC and test AUC values (large disparities indicate overfitting). Although a useful metric for evaluating threshold‐independent model performance, AUC is sensitive to the geographic extent of background sampling (Lobo et al. 2008). We reduced this source of bias by distributing background points similarly to presence points (Figure 3) and complemented model evaluation with threshold‐dependent metrics, described below.

We converted the cloglog output to a binary prediction of potential habitat (1 = habitat, 0 = nonhabitat) using the equal sensitivity and specificity threshold. We then assessed the fit of the full occurrence dataset and background points to the binary output using the true skill statistic (TSS) and the symmetric extremal dependence index (SEDI; Wunderlich et al. 2019). The TSS is commonly reported and does not depend on model prevalence, whereas the SEDI is less sensitive than the TSS for models built with many background points and low prevalence (Wunderlich et al. 2019). The TSS and model accuracy metrics range from 0 to 1 (1 = best model fit), and the SEDI ranges from −1 to 1, where values > 0 indicate a prediction better than random and values approaching 1 imply high predictive power. We additionally assessed model accuracy by comparing the number of correctly classified presence and background points to the total number of presence and background points. To determine which variables most influenced habitat predictions, we used the permutation importance and percent contribution outputs provided by Maxent. These metrics provide percentages ranging from 0% (least important) to 100% (most important).

### 2.6 Potential Habitat

The binary output produced from the equal sensitivity and specificity threshold provided a first step to identifying potentially suitable breeding habitat. To reduce the likelihood of underfitting and produce a better characterization of suitable habitat, we refined this output by bifurcating the cloglog values above the binary threshold into two classes using Jenks Natural Breaks (Jenks 1967) and only retaining pixels in the higher class. We additionally removed small patches (≤ 2 pixels) of isolated pixels (> 1 km from larger patches) to filter out functionally disconnected habitats. Finally, we removed pixels embedded within landscapes dominated by urban cover. To accomplish this, we computed the total area classified as developed (Developed—Low, Medium, and High Intensity, or Barren Land) within a 500‐m buffer around each pixel of predicted breeding habitat. If > 50% of the total buffer area was developed, we removed the habitat pixel.

In addition to identifying suitable breeding habitat, we extended the predicted area to include potential terrestrial habitat used by adults. To accomplish this, we buffered the refined breeding habitat by 500 m, based on observed adult space‐use patterns derived from radio telemetry (Flenniken, J. M. [2025], unpublished data), and extracted pixels representing potentially suitable terrestrial habitat for adults (Evergreen Forest, Shrub/Scrub, and Grassland/Herbaceous land cover classes with < 50% canopy cover). Collectively, these outputs represent a holistic model of breeding and terrestrial habitat for *Z. sayi* .

### 2.7 Potential Conservation Gaps

To identify potential conservation gaps for *Z. sayi* , we created a spatial layer combining contemporary protected areas (public and private) in Florida (Florida Natural Areas Inventory 2024), Georgia (Georgia Department of Natural Resources [DNR] 2024), and Alabama (USGS 2024b), and computed the percentage of the final model contained within those protected areas. Additionally, we used the NLCD and Landscape Fire and Resource Management Planning Tools (LANDFIRE 2024) to assess contemporary localized, indirect impacts from urban development, agriculture, and tree plantations adjacent to suitable habitat. To accomplish this, we computed their proportionate cover within a 60‐m (1 pixel) buffer of the final model.

To assess potential future impacts from urban development, we used the SLEUTH urban growth model modified for the Southeast Regional Assessment Project (SLEUTH Projected Urban Growth 2019). The SLEUTH model applies urban growth trajectories to predict the probability of future development adjacent to existing urban areas. We pooled the probability of urbanization into three classes (< 50%, ≥ 50%, and total) and computed the percentage of the final model projected to be developed by 2070. We omitted any urban projections that occurred within existing protected areas before we ran our analysis, which affected < 2% of projected urban area.

## 3 Results

### 3.1 Occurrence and Environmental Data

After cleaning and spatially thinning the 211 occurrences, we retained 112 (Florida = 76, Georgia = 36) nymph records and 20 (Florida = 17, Georgia = 3) adult records (moved downslope) for a total of 132 occurrences (Figure 2). The majority (85%) of the final occurrences were collected from 2013 to 2024. The mean ± SE nearest neighbor distance between occurrences was 4.4 ± 0.7 km, indicating a non‐localized distribution of samples.

Changes in land cover class were detected for 11 out of 132 occurrences (8%; Table 2). After cross‐referencing satellite imagery, only four of these changes were genuine (i.e., real changes in land cover not resulting from variances in mapping processes; Table 2). Over the entire study area, the mean ± SE change in percentage land cover from 2008 to 2024 was 0.6% ± 0.2%. The largest change (2.5%) was a reduction of 2979 km^2^ in Evergreen Forest (Table 3), which was primarily (78%) attributed to clearcutting of timber, resulting in conversion to Shrub/Scrub, Grassland/Herbaceous, or Barren Land. Land cover change was minimal from 2019 to 2024 (Table 3).

**TABLE 2:** Change in land cover class from 2008 to 2024 at the location of 11 occurrence records for *Zoraena sayi* . Land cover data were downloaded from the National Land Cover Database (NLCD) for 2008, 2019, and 2024. Each detected change in land cover class was cross‐referenced with satellite imagery (Google Earth Pro 7.3.7) to determine whether the change was genuine or resulted from variance in mapping processes.

Occurrence	State	NLCD 2008	NLCD 2019	NLCD 2024	Description	Change
2008	GA	Grassland/Herbaceous	Evergreen Forest	Evergreen Forest	Cleared timber early 2000	Genuine
2008	GA	Evergreen Forest	Grassland/Herbaceous	Shrub/Scrub	Cleared timber 2012 and growing in by 2024	Genuine
2012	FL	Woody Wetlands	Woody Wetlands	Grassland/Herbaceous	Cleared timber 2023; strip of Woody Wetlands remained	Genuine
2012	FL	Woody Wetlands	Woody Wetlands	Grassland/Herbaceous	Cleared timber 2023; strip of Woody Wetlands remained	Genuine
2015	FL	Cultivated Crops	Developed, Open Space	Developed, Open Space	Nearby road classified in later versions	Variance
2015	FL	Woody Wetlands	Evergreen Forest	Evergreen Forest	Noise; single pixel of Woody Wetlands in 2008	Variance
2015	FL	Shrub/Scrub	Evergreen Forest	Evergreen Forest	Surrounding Shrub/Scrub classified in 2008	Variance
2012	FL	Evergreen Forest	Evergreen Forest	Grassland/Herbaceous	Edge of class error; cleared land nearby	Variance
2011	FL	Evergreen Forest	Shrub/Scrub	Evergreen Forest	Edge of class error; cleared land nearby	Variance
2015	FL	Developed, Open Space	Evergreen Forest	Evergreen Forest	Platted 1980s but undeveloped; noise from single pixels	Variance
2015	FL	Developed, Open Space	Woody Wetlands	Woody Wetlands	Platted 1980s but undeveloped; noise from single pixels	Variance

**TABLE 3:** Change in land cover class from 2008 to 2024 and from 2019 to 2024 across the known range of *Zoraena sayi* (+10 km). Land cover data were downloaded from the National Land Cover Database (NLCD) for 2008, 2019, and 2024.

Class	Area km^2^			Change 2008–2024		Change 2019–2024	
	2008	2019	2024	km^2^	%	km^2^	%
Open Water	1785	1866	1850	65	0.1	−16	0.0
Developed, Open Space	6330	6476	6720	390	0.3	244	0.2
Developed, Low Intensity	3084	3259	3365	281	0.2	106	0.1
Developed, Medium Intensity	641	730	781	140	0.1	51	0.0
Developed, High Intensity	156	181	195	39	0.0	14	0.0
Barren Land	347	366	480	133	0.1	114	0.1
Deciduous Forest	254	239	236	−19	0.0	−3	0.0
Evergreen Forest	38,500	35,759	35,521	−2979	−2.5	−238	−0.2
Mixed Forest	846	648	590	−257	−0.2	−58	0.0
Shrub/Scrub	4270	5065	5457	1187	1.0	392	0.3
Herbaceous	4830	6665	6409	1579	1.3	−256	−0.2
Hay/Pasture	7140	7527	7826	687	0.6	299	0.3
Cultivated Crops	17,730	17,426	16,885	−845	−0.7	−541	−0.5
Woody Wetlands	30,831	30,611	30,025	−806	−0.7	−586	−0.5
Emergent Herbaceous Wetlands	868	794	1271	403	0.3	477	0.4

### 3.2 Model Performance

The optimal (lowest AICc) model parameters were a regularization multiplier of 1 and LQHP feature classes. The mean ± SD training AUC (0.98 ± 0.00) and test AUC (0.97 ± 0.01) were considered outstanding and not overfitted to the data based on their similarity. Evaluations of the data fit to the binary output (equal sensitivity and specificity threshold = 0.098) were strong with a TSS of 0.88, SEDI of 0.96, and accuracy of 0.95.

**FIGURE 4:**
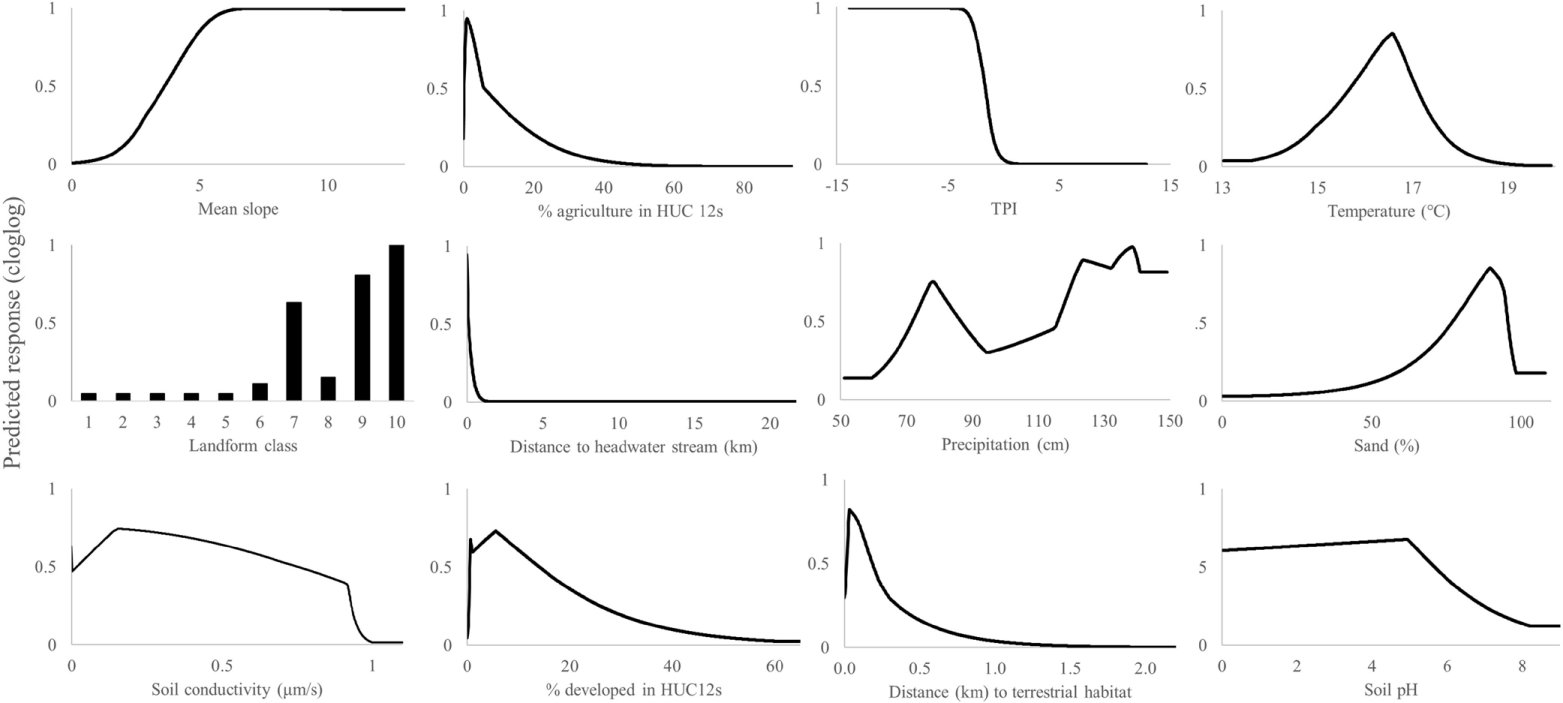
Response curves generated by Maxent for environmental variables used to model breeding habitat (1 = habitat, 0 = non‐habitat) for *Zoraena sayi* in the southeastern United States. The model was built using linear, quadratic, hinge, product, and categorical feature classes.

The variables that most influenced (permutation importance or percent contribution values ≥ 10) the model were mean slope and percent agriculture within subwatersheds, followed by the TPI, mean March temperature, and landform class (Table 4). Response curves (Figure 4) indicated that higher slope values (relatively steeper areas) and lower percent agriculture were associated with higher habitat suitability, as were negative TPI values (valleys, depressions), intermediate March temperatures, and landform classes 7, 9, and 10 (low depression classes).

**TABLE 4:** Relative influence of environmental variables used to model breeding habitat for *Zoraena sayi* in the southeastern United States. Variable influence was based on mean percent permutation importance and percent contribution from 10‐fold cross validated model runs in Maxent. Variables with mean values ≥ 10% were considered most influential.

Variable	Permutation importance		Percent contribution	
	Mean	SD	Mean	SD
Slope	27	3	23	11
% Agriculture	26	5	16	4
TPI	6	1	26	18
Temperature (March)	11	3	4	2
Landform	1	< 1	11	2
Distance to stream	9	3	8	1
Precipitation (March)	8	1	3	1
% Sand	6	1	3	2
Conductivity	5	1	5	1
% Developed	1	< 1	1	< 1
Distance to terrestrial habitat	< 1	< 1	< 1	< 1
pH	< 1	< 1	< 1	1

### 3.3 Potential Habitat

**FIGURE 5:**
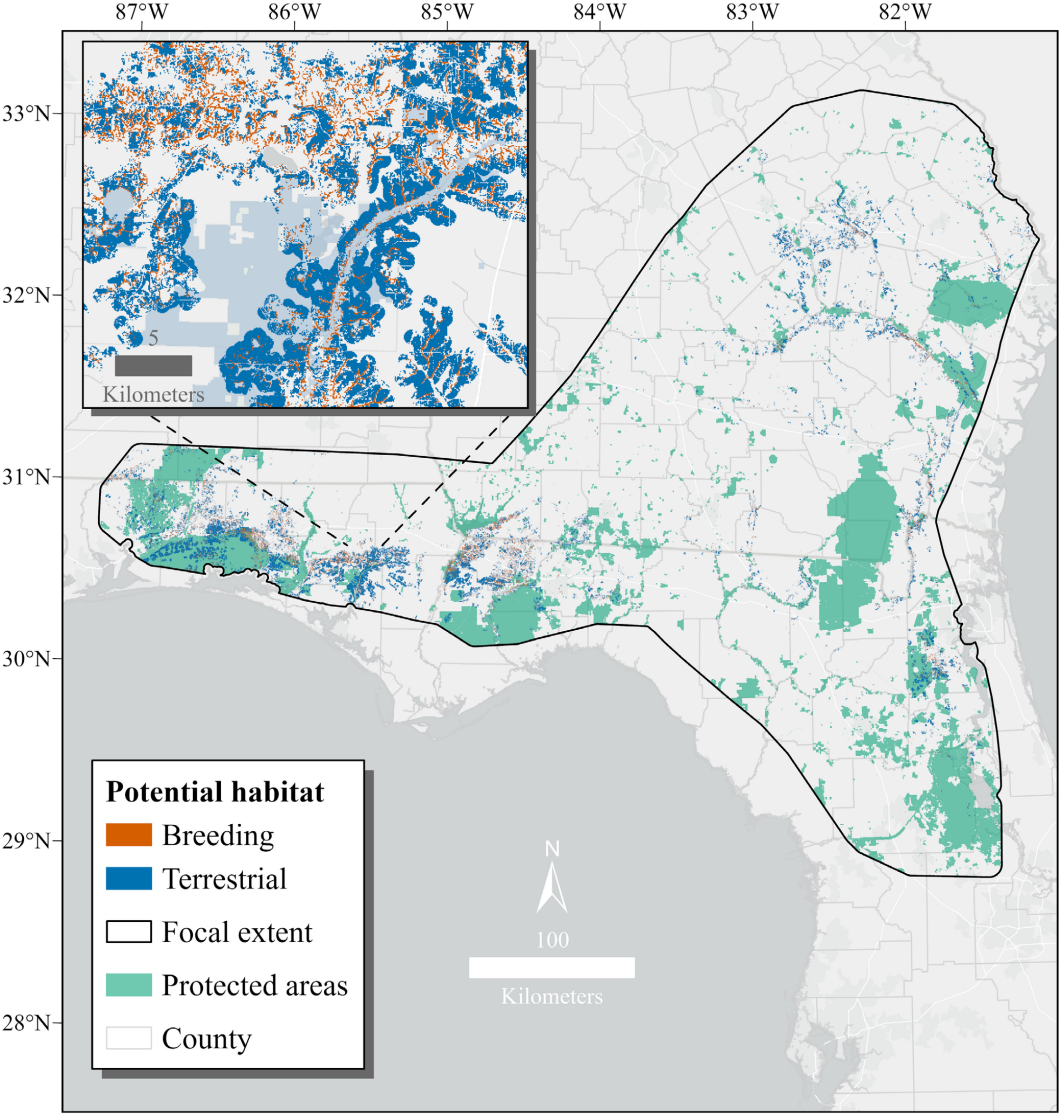
Potential breeding and terrestrial habitat for *Zoraena sayi* modeled using occurrence data collected from 2008 to 2024 and environmental data characterizing climate, land cover, soil, and topography at local, catchment, and regional scales. Focal extent was based on estimated *Z. sayi* range from Paulson (2011) plus a 10 km buffer. Protected areas are lands acquired by government (federal, state, or local) or private entities for conservation purposes.

The total area of predicted breeding habitat produced by the binary threshold was 2212 km^2^. After refining the binary output, 502 km^2^ of suitable habitat remained. The estimated area of terrestrial habitat was 2229 km^2^ for a combined area of 2731 km^2^ represented by the final model (Figure 5). Of the final model area, 69% occurred in Florida, 29% in Georgia, and 3% in Alabama. The largest concentrations of suitable habitat occurred throughout the panhandle region of Florida.

### 3.4 Potential Conservation Gaps

Contemporary protected areas encompass 30% (819 km^2^) of suitable habitat represented by the final model (Table 5), meaning 70% (1912 km^2^) of suitable habitat is not currently protected. Only 13% of modeled habitat is indirectly threatened by adjacent agriculture or developed lands, but 36% is potentially threatened by neighboring tree plantations (Table 5). Of the unprotected habitat, 50% (956 km^2^) is predicted to be developed by 2070 according to the SLEUTH model (Table 5). Despite having the highest proportion of protected habitat (37%; 693 km^2^) compared to other states, Florida also has the highest proportion of habitat threatened by future development (41%; 768 km^2^). Potential conservation gaps and impacts of future urbanization are similar for breeding and terrestrial habitat regardless of state. For example, 31% of breeding habitat is protected in Florida compared to 38% of terrestrial habitat, a difference of 7%.

**TABLE 5:** Proportion of suitable habitat (breeding and terrestrial) for *Zoraena sayi* occurring within protected area boundaries, or that is potentially threatened by adjacent plantation, agriculture, or developed lands. Suitable habitat threatened by future urbanization was based on areas projected to be developed by 2070 (SLEUTH Projected Urban Growth). Protected area boundaries were produced as of 2025 for Georgia and 2024 for Florida and Alabama. Urban growth projections were divided into two classes based on the probability of development.

Category	Florida		Georgia		Alabama		Total	
	%	km^2^	%	km^2^	%	km^2^	%	km^2^
Protected areas	37	693	14	110	27	19	30	819
Plantations	31	580	45	354	62	45	36	983
Agriculture	6	112	12	94	7	5	8	218
Development	5	94	6	47	4	3	5	137
Projected development								
Probability < 50%	9	168	7	55	4	3	8	218
Probability ≥ 50%	32	599	21	165	4	3	27	737
Total	41	768	28	220	8	6	35	956

## 4 Discussion

We modeled potential habitat for *Z. sayi* across the southeastern United States using a combination of nymph (85%) and adult (15%) occurrence data. By including adult occurrences (largely from community science databases) we increased the sample size and geographic representation, both of which can improve the performance of SDMs for rare or range‐restricted species (Wisz et al. 2008; Williams et al. 2009). Through our multi‐scale modeling approach, we found that steep ravines located within subwatersheds with minimal agriculture were most suitable for *Z. sayi* (Table 4), which is largely consistent with field observations of breeding habitat. While not suitable for breeding, terrestrial habitats are important for adult foraging, thermoregulation, and dispersal (Dolný et al. 2014; Minot et al. 2021). We modeled this ecological linkage by explicitly incorporating predictor variables relevant to adults. Mean temperature in March (when most adults emerge) was among the more influential variables in our model (Table 4), which demonstrates the value of considering adult traits in model development. By estimating potential terrestrial habitat within a 500‐m buffer of predicted breeding habitat, we modeled the full spectrum of aquatic and terrestrial habitats required by *Z. sayi* to enable a comprehensive analysis of current protections and future threats to suitable habitat throughout the southeastern United States (Table 5).

Fittingly, our model corroborated prior research that identified the Florida panhandle (Keppner 2013) and the Vidalia Uplands physiographic province in southeastern Georgia (Stevenson et al. 2009) as significant regions for *Z. sayi* occupancy. Additionally, suitable habitat was predicted in unsampled areas, such as on private, unprotected lands throughout the Florida panhandle. These areas represent prospective opportunities for conservation efforts via land acquisition should they be occupied or within the dispersal range of occupied sites, especially considering the high probability of future urbanization in the region. A small area of suitable habitat was also predicted to occur along the Suwannee River in Hamilton County, Florida, which represents a regional gap in the known distribution of *Z. sayi*. Efforts to document the species from this region have thus far been unsuccessful (J. D. Mays, personal communication), but additional surveys may be warranted given our model predictions and historical records of *Z. sayi* from adjacent Columbia County, FL in 1896–1897 (Westfall 1953). Alternatively, the lack of available occurrence data from this region combined with our model predictions may suggest a natural barrier to dispersal that has created disjunct eastern and western populations; however, additional research into population genetic structure would be necessary to support this hypothesis. It is important to emphasize that our model estimates potentially suitable habitat but does not signify occupancy or convey the status of existing populations. This distinction should be made transparent when our model is applied to conservation planning in order to effectively allocate limited resources (Loiselle et al. 2003). To this end, site evaluations and occupancy surveys should be conducted by species experts to prioritize modeled habitat areas and thereby maximize conservation impact. Regardless, our model can serve as a valuable tool for guiding future surveys and identifying opportunities for conservation.

The International Union for Conservation of Nature cites residential and commercial development as a primary factor threatening *Z. sayi* populations (Paulson 2018). In Florida, where we predict 69% of suitable habitat to occur, the human population is projected to exceed 33 million by 2070—an increase of 15 million from 2010 (Florida Bureau of Economic and Business Research 2015). Our analysis suggests that the majority (70%) of habitat potentially suitable for *Z. sayi* is not safeguarded by contemporary protected areas, and that approximately half of this unprotected habitat (35% of total habitat) may be impacted by urbanization by 2070. Moreover, while it is beyond the scope of this study to estimate the extent of suitable habitat already lost to destruction and degradation, at least one *Z. sayi* population has disappeared from Florida in recent decades following increases in residential development (Mauffray 1995). Even potentially suitable habitat located within protected areas could be indirectly impacted (e.g., via runoff or pollution) by adjacent land uses or directly affected within areas that are designated for multiple uses and contain silviculture or ranching lands. With > 30% of modeled habitat located adjacent to tree plantations (Table 5), there is potential for indirect negative impacts to *Z. sayi* populations. In a multi‐species assessment of riverine dragonflies in South Africa, Samways and Steytler (1996) recommend establishing riparian buffers of 20–30 m to limit negative impacts to breeding sites from commercial silviculture operations; however, this recommendation alone is likely inadequate to protect *Z. sayi* populations due to their sensitivity to catchment‐scale influences and extensive use of terrestrial landscapes hundreds of meters away from breeding sites (Flenniken, J. M. [2025], unpublished data). Protecting suitable habitat from future development and encouraging best management practices, such as selective or reduced‐impact logging within silviculture operations near breeding habitat (Stevenson et al. 2009; Calvão et al. 2016; Luke et al. 2017), are likely the best courses of action to preserve remaining *Z. sayi* populations.

Our analysis makes use of the best available data to evaluate current protections and future threats to suitable habitat for *Z. sayi* throughout their range in the southeastern United States. We estimate that more than a third of suitable habitat is not covered by existing protected areas and is likely to experience impacts from urbanization by the year 2070. To effectively safeguard suitable habitat and preserve remaining *Z. sayi* populations, targeted land acquisition may be advisable, contingent upon the results of site evaluations and occupancy surveys conducted by species experts. This may be especially impactful in areas such as the Florida panhandle, where suitable habitat is seemly abundant, but protections are limited and future threats are significant. On multi‐use properties, the adoption of reduced‐impact silvicultural practices combined with large riparian buffers (e.g., > 100 m) and active fire management may help to mitigate indirect impacts on breeding sites and maintain suitable terrestrial habitat for adults.

## Author Contributions

**J. Matthew Flenniken:** conceptualization (lead), data curation (equal), methodology (supporting), writing – original draft (lead), writing – review and editing (lead). **Mark A. Barrett:** conceptualization (supporting), data curation (equal), formal analysis (lead), methodology (lead), visualization (lead), writing – original draft (supporting), writing – review and editing (supporting). **Dirk J. Stevenson:** conceptualization (supporting), data curation (equal), writing – review and editing (supporting).

## Funding

This work was supported by Fish & Wildlife Foundation of Florida, CWT25‐07 and Florida Fish and Wildlife Conservation Commission.

## Conflicts of Interest

The authors declare no conflicts of interest.

## Data Availability

The data, metadata, and code that support the findings of this study are openly available on GitHub and are archived on Zenodo at https://doi.org/10.5281/zenodo.19485345. Due to legal restrictions associated with the sharing of biologically sensitive data, occurrence records provided by the Florida Natural Areas Inventory and Georgia Department of Natural Resources have been removed from the archived dataset; the full dataset was made available for peer review.
